# Machine-Learning Application for Predicting Metabolic Dysfunction-Associated Steatotic Liver Disease Using Laboratory and Body Composition Indicators

**DOI:** 10.34172/aim.31269

**Published:** 2024-10-01

**Authors:** Fatemeh Masaebi, Mehdi Azizmohammad Looha, Morteza Mohammadzadeh, Vida Pahlevani, Mojtaba Farjam, Farid Zayeri, Reza Homayounfar

**Affiliations:** ^1^Department of Biostatistics, School of Allied Medical Sciences, Shahid Beheshti University of Medical Sciences, Tehran, Iran; ^2^Basic and Molecular Epidemiology of Gastrointestinal Disorders Research Center, Research Institute for Gastroenterology and Liver Diseases, Shahid Beheshti University of Medical Sciences, Tehran, Iran; ^3^Department of Biostatistics, School of Public Health, Iran University of Medical Sciences, Tehran, Iran; ^4^Department of Biostatistics, Faculty of Medical Science, Tarbiat Modares University, Tehran, Iran; ^5^Noncommunicable Diseases Research Center, Fasa University of Medical Sciences, Fasa, Iran; ^6^Proteomics Research Center and Department of Biostatistics, School of Allied Medical Sciences, Shahid Beheshti University of Medical Sciences, Tehran, Iran; ^7^National Nutrition and Food Technology Research Institute, Shahid Beheshti University of Medical Sciences, Tehran, Iran

**Keywords:** Logistic regression, Machine learning, Non-alcoholic fatty liver disease, Predictive models, Rural area

## Abstract

**Background:**

Metabolic dysfunction-associated steatotic liver disease (MASLD) represents a significant global health burden without established curative therapies. Early detection and preventive strategies are crucial for effective MASLD management. This study aimed to develop and validate machine-learning (ML) algorithms for accurate MASLD screening in a geographically diverse, large-scale population.

**Methods:**

Data from the prospective Fasa Cohort Study, initiated in rural Fars province, Iran (March 2014), were employed for this purpose. The required data were collected using blood tests, questionnaires, liver ultrasonography, and physical examinations. A two-step approach identified key predictors from over 100 variables: (1) statistical selection using mean decrease Gini in random forest and (2) incorporation of clinical expertise for alignment with known MASLD risk factors. The hold-out validation approach (with a 70/30 train/validation split) was utilized, along with 5-fold cross-validation on the validation set. Logistic regression, Naïve Bayes, support vector machine, and light gradient-boosting machine (LightGBM) algorithms were compared for model construction with the same input variables based on area under the receiver operating characteristic curve (AUC), sensitivity, specificity, positive predictive value (PPV), negative predictive value (NPV), and accuracy.

**Results:**

A total of 6,180 adults (52.7% female) were included in the study, categorized into 4816 non-MASLD and 1364 MASLD cases with a mean age (±standard deviation [SD]) of 48.12 (±9.61) and 49.47 (±9.15) years, respectively. Logistic regression outperformed other ML algorithms, achieving an accuracy of 0.88 (95% confidence interval [CI]: 0.86-0.89) and an AUC of 0.92 (95% CI: 0.90-0.93). Among more than 100 variables, the key predictors included waist circumference, body mass index (BMI), hip circumference, wrist circumference, alanine aminotransferase levels, cholesterol, glucose, high-density lipoprotein, and blood pressure.

**Conclusion:**

Integration of ML in MASLD management holds significant promise, particularly in resource-limited rural settings. Additionally, the relative importance assigned to each predictor, particularly prominent contributors such as waist circumference and BMI, offers valuable insights into MASLD prevention, diagnosis, and treatment strategies.

## Introduction

 Metabolic dysfunction-associated steatotic liver disease (MASLD) is a common chronic liver condition that affects people worldwide. It is characterized by the build-up of excess fat in liver cells, regardless of alcohol consumption.^1,2^ The global incidence of MASLD is estimated to be 47 cases per 1000 people, with a prevalence of approximately 30% among adults. The trend analysis of epidemiological studies has revealed a consistent increase in the global prevalence of MASLD, rising from 26% in 2005 to 38% in 2016. The prevalence of MASLD varies significantly across regions and is influenced by factors such as socioeconomic status and obesity rates. A newly published paper has reported that the prevalence of MASLD has soared to over 35% among the Iranian population.^3^ If this trend remains unchanged, the global prevalence of MASLD in 2040 is expected to exceed 50.0%, with a yearly increase of 2.16% from 2020 to 2040.^4-6^

 In terms of clinical indicators, it is evident that a sedentary lifestyle significantly accelerates the progression of MASLD. It is considered the root cause of a range of associated risk factors, including obesity, hypertension, high cholesterol levels, metabolic syndrome, high wrist and hip circumference, type 2 diabetes, and insulin resistance.^7-11^ However, it should be noted that these indicators can also be influenced by genetic factors.^12^ In this context, recognition of the mentioned risk factors and early detection of MASLD are pivotal in preventing and timely managing the disease.

 The MASLD can be diagnosed through noninvasive or invasive methods. First, blood tests, along with a medical history, are used to evaluate liver function.^13^ Imaging tests and magnetic resonance imaging are other noninvasive methods that provide detailed images to identify the presence of fatty deposits.^14^ Additionally, the fatty liver index, calculated based on body mass index (BMI), waist circumference, triglyceride levels, and gamma-glutamyl transferase levels, is a numerical scoring tool commonly used to detect individuals with MASLD.^15^ In terms of invasive methods, liver biopsy is considered a gold standard for detecting MASLD and evaluating the severity of liver damage. However, liver biopsy is typically recommended in situations where additional information is needed for treatment decisions.^16^ In addition to these clinical methods, several statistical approaches have been recently suggested by data analysts utilizing different indicators for predicting MASLD. These models often utilize biomarkers, machine-learning (ML) techniques, and anthropometric indices. Some models focus on urinary protein panels or specific genetic markers to predict the risk of MASLD.^17-19^ External validation of these models is crucial to ensure their accuracy and applicability across different populations.^20,21^

 Current diagnostic methods for MASLD face significant limitations that hinder their effectiveness in clinical practice. Invasive procedures such as liver biopsy, despite being the gold standard, are associated with risks such as pain, bleeding, and variability in sampling, leading to low patient acceptance and potential diagnostic inconsistencies.^22^ Noninvasive methods, including imaging tests and biochemical markers, often lack the necessary sensitivity and specificity, resulting in potential misdiagnoses.^23^ Emerging biomarker-based models show promise but require further validation to be reliably integrated into routine use.^24^

 In recent years, there has been a growing application of ML in the field of medicine to build disease prediction models and classify patients with or without disease.^25^ This approach is capable of performing tasks that traditionally require human abilities such as learning, reasoning, and perception. ML involves the use of algorithms to predict unknown information by learning the inherent statistical patterns and structures within data. In the analysis of large bulks of data, ML outperforms traditional statistical methods by its ability to accurately identify variables with a significant impact on medical outcomes. Additionally, ML exhibits superior predictive performance and excels in modeling complex relationships between variables. It can learn from multiple sources of data, enabling a more comprehensive analysis. Moreover, ML techniques demonstrate robustness, even in the presence of data noise.^26^

 Regarding the appealing features of ML, it is actively utilized to construct models for identifying and classifying subjects with or without MASLD. For instance, a 2021 study by Atsawarungruangkit et al evaluated twenty-four ML methods among the American population and finally recommended the use of simpler models, such as coarse trees, which offer tangible interpretation and are easy to use in clinical practice. The majority of studies conducted in this field have mainly focused on laboratory indicators, while only a small number have considered body composition factors.^27-29^ Another study performed on a sample of 513 Iranian subjects reported that the random forest (RF) algorithm, utilizing body factors, provides the most accurate prediction model for MASLD.^30^ Regarding the lack of adequate information in this field, the current study was conducted to evaluate the performance of ML methods on a large-scale Iranian population, considering both body composition and laboratory indicators simultaneously, to identify the best classifier for MASLD. The study also seeks to investigate the effect of influential factors on MASLD based on an interpretable ML method.

## Materials and Methods

###  Study Design 

 The current study is a part of the baseline phase of the prospective Fasa Cohort Study, initiated at the Fasa University of Medical Sciences in March 2014. This study was designed to identify influential indicators of MASLD and accurately predict and classify individuals with and without MASLD within the rural population of the Fasa region, utilizing ML models.

###  Sampling Technique

 The Fasa Cohort Study specifically targets rural areas in Fars Province, located in the southern part of Iran, known as Nobandagan, Sheshdeh, Qarabolagh, and Shibkuh, with a total population of approximately 205 000 individuals. Firstly, the random sampling method was employed to select the areas, and then from these selected areas, namely, Sheshdeh and Qarabolagh, the census approach was utilized to choose the participants. Secondly, the information about the residents and their willingness to participate in the cohort study was obtained through the cooperation of the health vice-chancellors of Fasa University and the local health workers in each village. The inclusion criteria required participants to be Iranian nationals, have resided in the area for at least one year, be between 35 and 70 years old, and have effective verbal communication skills.^31,32^

###  Participants and Study Setting

 The enrollment phase of FACAS started from October 2014 to September 2016 in the Sheshdeh and Qarabolagh areas of Fasa, and four phases have been conducted until the end of 2021. In these areas, 11 097 individuals between the ages of 35 and 70 were identified as eligible; among them, 10 118 were willing to participate in the study.

**Figure 1:**
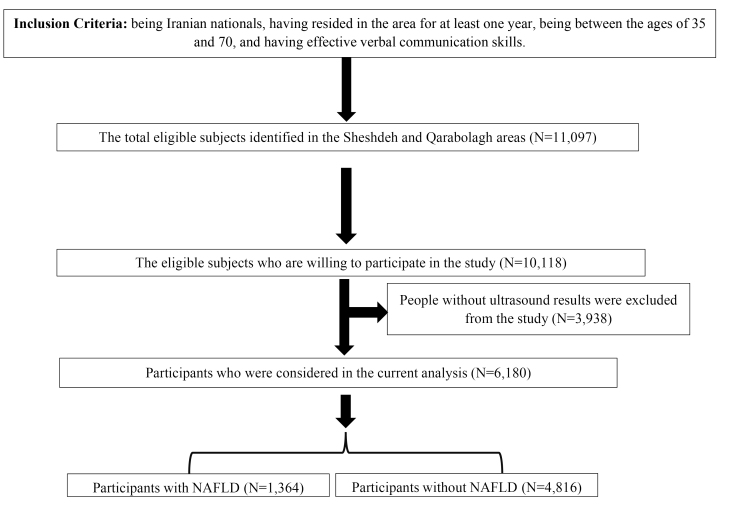
The Flow Chart of Enrolling Participants in the Current Study

 The participants had a 25-mL blood sample taken for laboratory examinations, which was subsequently transferred to the Cohort Reference Laboratory at Fasa University of Medical Sciences. They also underwent clinical examinations and questionnaire surveys administered by trained interviewers. These interviewers utilized digital data acquisition sheets, as well as valid and standardized questionnaires and screening tools, to collect information. The interviewers were categorized into general, medical, and nutrition roles based on the specific type of questionnaire they managed.^33,34^ Ultimately, the liver status of each participant was determined based on sonographic evidence and reviewing the medical history of patients. This enabled volunteers who had recently undergone ultrasound and received confirmed diagnoses of MASLD by physicians to participate in the study. Consequently, a total sample of 6,180 individuals was included in the study. The process of enrolling participants in the current study is illustrated in Figure 1.

###  Variables Under Study

 The primary outcome variable under investigation was the presence or absence of MASLD, determined through ultrasonography examinations and confirmed diagnoses by physicians.

 In terms of explanatory variables (predictors), the original data set for this study included 400 variables. However, to ensure the accuracy and relevance of the analysis, variables with 40% or more missing data were excluded, resulting in a final set of 380 variables. Next, variables that were unlikely to be relevant to MASLD, those that were considered to be outcomes of MASLD, and variables that provided redundant information (e.g., both continuous and categorized blood pressure) were removed from consideration as explanatory variables. The remaining variables were then analyzed using the independent samples t-test and Chi-square test for continuous and categorical predictors, respectively, to determine if they show any significant difference between those with and without MASLD. As a result, 103 variables demonstrated statistically significant relationships with the outcome variable at the 20% level of significance (*P* < 0.1, Table 1). The methodology for obtaining each variable is detailed in Table S1 in Supplementary file 1.

**Table 1:** Clinical Parameters Based on Subjects With and Without MASLD

**Variables**	**No MASLD (n=4816)**	**MASLD (n=1364)**	***P*** **-value**
**Categorial variables**	**No. (%)**	**No. (%)**	
Anti-DM-pill (Yes)	106 (2.20)	159 (11.70)	< 0.001
Anti-HTN-drug (Yes)	369 (7.70)	307 (22.50)	< 0.001
FH1_diabetes (Yes)	1253 (26.00)	565 (41.40)	< 0.001
FH1-hypertension (Yes)	2346 (48.70)	838 (61.40)	< 0.001
FH2-diabetes (Yes)	777 (16.10)	319 (23.40)	< 0.001
FH2_hypertension (Yes)	753 (15.60)	275 (20.20)	0.013
Gender (Male)	2290 (47.50)	965 (70.70)	< 0.001
Has diabetes (Yes)	361 (7.50)	316 (23.20)	< 0.001
Has gallstone (Yes)	76 (1.60)	85 (6.20)	< 0.001
Has learning disability (Yes)	516 (10.70)	105 (7.70)	< 0.001
Has osteoporosis disease (Yes)	395 (8.20)	209 (15.30)	< 0.001
Has rheumatic disease (Yes)	191 (4.00)	95 (7.00)	< 0.001
Has shortness of breath (Yes)	211 (4.40)	102 (7.50)	< 0.001
Has sternum irritation (Yes)	1105 (22.90)	746 (27.70)	< 0.001
Has swelling (Yes)	677 (14.10)	460 (33.70)	< 0.001
Has thyroid disease (Yes)	352 (7.30)	179 (13.10)	0.003
Has weight loss (Yes)	111 (2.30)	19 (1.40)	< 0.001
Smoking (Yes)	1601 (33.20)	739 (54.20)	< 0.001
TG-lowering-drug (Yes)	13 (0.30)	27 (2.00)	< 0.001
Using alcohol (Yes)	277 (5.80)	41 (3.00)	< 0.001
Using insulin (Yes)	13 (0.30)	14 (0.10)	0.002
Using statins (Yes)	192 (4.00)	166 (12.20)	< 0.001
**Continuous variables**	**Mean±SD**	**Mean±SD**	
Age (years)	48.12 ± 9.61	49.47 ± 9.15	< 0.001
Alanine (g/d)	2.49 ± 1.23	2.40 ± 1.15	0.007
ALP (IU/L)	202.88 ± 64.55	217.69 ± 86.74	< 0.001
Alpha carotene (µ/d)	779.99 ± 866.48	827.58 ± 883.19	0.003
Alpha-linolenic acid (g/d)	7.81 ± 6.68	7.80 ± 5.55	< 0.001
Arginine (g/d)	3.01 ± 1.48	3.09 ± 1.55	0.006
Aspartic Acid (g/d)	5.11 ± 2.36	4.98 ± 2.26	< 0.001
Beta carotene (mg/d)	4863.81 ± 3547.24	5459.25 ± 3820.11	< 0.001
BMI (kg/m^2^)‎	22.72 ± 4.02	30.45 ± 4.58	< 0.001
BUN (mg/dL)	13.18 ± 4.12	12.54 ± 3.69	< 0.001
Caffeine (mg/d)	200.85 ± 201.09	166.67 ± 153.56	< 0.001
CHOL (mg/dL)	176.85 ± 36.30	196.69 ± 40.63	< 0.001
Cholesterol (mg/d)	276.76 ± 166.45	242.86 ± 151.15	< 0.001
Cryptoxanthin, beta (µ/d)	250.11 ± 243.23	286.83 ± 261.83	< 0.001
DBP (mmHg)	71.97 ± 11.03	77.28 ± 12.05	< 0.001
Docosahexaenoic acid (g/d)	0.02 ± 0.03	0.03 ± 0.02	0.003
Eicosapentaenoic acid (g/d)	0.46 ± 0.32	0.45 ± 0.33	0.005
Fatty acid (total saturated) (g/d)	25.43 ± 12.23	22.59 ± 10.90	< 0.001
Fatty acid (total trans) (g/d)	0.34 ± 0.39	0.28 ± 0.41	< 0.001
Fatty acids, MUFA (g/d)	19.06 ± 9.21	17.34 ± 8.42	< 0.001
Fiber (total) (g/d)	30.71 ± 13.84	30.74 ± 13.16	< 0.001
Fluoride (µ/d)	3717.20 ± 3732.40	3100.02 ± 2857.92	< 0.001
Fructose (g/d)	29.57 ± 19.90	32.88 ± 21.09	< 0.001
GLUC (mg/dL)	88.71 ± 23.18	99.57 ± 37.14	< 0.001
Glucose (dextrose) (g/d)	24.62 ± 19.09	32.45 ± 21.60	< 0.001
Glutamic acid (g/d)	9.38 ± 4.25	9.17 ± 4.06	0.002
Glycine (g/d)	1.98 ± 1.02	1.90 ± 0.94	0.011
GR (%)	54.65 ± 11.03	53.22 ± 10.18	0.006
HCT (g/dL)	41.81 ± 4.38	41.96 ± 4.32	< 0.001
HDLC (mg/dL)	52.48 ± 16.22	48.09 ± 13.62	< 0.001
HGB (g/dL)	14.68 ± 1.67	14.58 ± 1.61	0.003
Hip circumference (cm)	94.86 ± 7.24	107.40 ± 9.22	< 0.001
Histidine (g/d)	1.29 ± 0.65	1.25 ± 0.61	0.012
Isoleucine (g/d)	2.27 ± 1.11	2.20 ± 1.03	0.009
LDL (mg/dL)	103.63 ± 30.91	113.48 ± 33.87	< 0.001
Leucine (g/d)	3.78 ± 1.84	3.64 ± 1.70	0.021
Lutein + zeaxanthin (µ/d)	2921.66 ± 3307.36	3207.40 ± 2381.56	< 0.001
LY (%)	42.21 ± 10.40	43.50 ± 9.54	0.018
Lycopene (µ/d)	14248.87 ± 8889.18	15451.65 ± 10306.89	< 0.001
Lysine (g/d)	3.33 ± 1.71	3.23 ± 1.58	0.004
Magnesium (mg/d)	394.89 ± 153.53	381.68 ± 144.26	0.002
MCH (Pg)	29.90 ± 3.38	29.33 ± 3.13	< 0.001
MCHC (Gr/dL)	35.08 ± 1.44	34.77 ± 1.59	< 0.001
MCV (FL)	85.07 ± 8.07	84.30 ± 7.52	0.026
MET (h/d)	43.38 ± 12.51	39.10 ± 9.48	< 0.001
Methionine (g/d)	1.08 ± 0.55	1.04 ± 0.51	0.01
MO (%)	3.14 ± 1.34	3.27 ± 1.38	< 0.001
n-3 (total) (g/d)	0.04 ± 0.03	0.04 ± 0.03	0.097
n-6 (total) (g/d)	4.66 ± 3.37	4.86 ± 3.39	0.019
Naps (hour)	0.77 ± 0.87	0.92 ± 0.91	< 0.001
Night sleep (hour)	6.97 ± 1.62	6.75 ± 1.62	< 0.001
Pantothenic acid (mg/d)	5.26 ± 2.34	5.27 ± 2.33	< 0.001
Phenylalanine (g/d)	2.23 ± 1.05	2.15 ± 0.98	0.018
PLT (Cumm)	264.87 ± 68.43	291.42 ± 77.04	< 0.001
Potassium (mg/d)	3850.12 ± 1581.12	3884.45 ± 1610.46	< 0.001
Proline (g/d)	2.72 ± 1.32	2.64 ± 1.22	0.018
RBC (Cumm)	4.94 ± 0.59	5.03 ± 0.66	< 0.001
Retinol (µ/d)	357.37 ± 289.02	302.18 ± 227.11	< 0.001
SBP (mmHg)	107.37 ± 17.33	115.61 ± 18.26	< 0.001
Selenium (µ/d)	152.08 ± 76.44	135.97 ± 67.51	0.012
Serine (g/d)	2.39 ± 1.12	2.30 ± 1.04	0.047
SGOT (IU/L)	21.92 ± 7.35	23.91 ± 10.98	< 0.001
SGPT (IU/L)	21.24 ± 7.34	28.24 ± 17.28	< 0.001
Sugar (g/d)	174.96 ± 94.82	160.60 ± 83.44	0.053
TG (Mg/dL)	127.09 ± 80.29	134.93 ± 83.47	< 0.001
Threonine (g/d)	1.97 ± 0.96	1.90 ± 0.89	0.012
Total lipid (g/d)	71.95 ± 30.29	65.24 ± 27.29	< 0.001
Tryptophan (g/d)	0.55 ± 0.26	0.54 ± 0.24	0.013
Tyrosine (g/d)	1.66 ± 0.81	1.61 ± 0.75	0.015
Valine (g/d)	2.69 ± 1.27	2.61 ± 1.19	0.007
Vitamin A (µ/d)	807.84 ± 502.14	805.16 ± 474.36	0.001
Vitamin B6 (mg/d)	11.02 ± 8.65	10.99 ± 7.23	< 0.001
Vitamin C (mg/d)	139.98 ± 92.71	153.33 ± 106.23	< 0.001
Vitamin E (mg/d)	8.28 ± 4.02	8.26 ± 4.01	< 0.001
Vitamin K (µ/d)	191.57 ± 176.73	214.41 ± 152.04	< 0.001
Waist circumference (cm)	85.76 ± 9.88	104.76 ± 10.41	< 0.001
WBC (Cumm)	6.32 ± 1.74	6.79 ± 1.86	< 0.001
Wrist circumference (cm)	16.24 ± 1.20	17.46 ± 1.42	< 0.001
WSI-total	-0.96 ± 0.87	-0.72 ± 0.83	< 0.001

*Note*. MASLD: Metabolic dysfunction-associated steatotic liver disease; SD: Standard deviation; DM: *Diabetes mellitus*; HTN: *Hypertension*; FH1-diabetes: Fumarate hydratase 1; FH1-hypertension: Familial hyperaldosteronism type 1; ALP: *Alkaline phosphatase*; BMI: Body mass index; BUN: Blood urea nitrogen; CHOL: Cholesterol; DBP: Diastolic blood pressure; MUFA: Monounsaturated *fatty acids*; GR: Granulocyte; HCT: *Hematocrit*; HDLC: High-density lipoprotein cholesterol; HGB: *Hemoglobin*; LDL: Low-density lipoprotein; LY: *Lycopene*; MCH: *Mean* corpuscular hemoglobin; MCHC: *Mean* corpuscular hemoglobin concentration; MCV: *Mean corpuscular volume*; MET: *Met* abolic equivalent of task; MO: monocytes; PLT: *Platelet*; RBC: Red blood cell; SBP: Systolic blood pressure; SGOT: Serum glutamic oxaloacetic transaminase; SGPT: Serum glutamate pyruvate transaminase; TG: *Triglyceride*; WBC: *White blood cell count*; WSI: wealth score index; GLUC: Glucose.

###  Statistical Analysis

 Descriptive data were presented by categorizing patients based on their risk of MASLD and analyzing their features. Categorical and numerical data were described as frequencies (percentages) and means ± standard deviations (SD). The analyses were performed using the R software (version 4.2.1). *P*-values less than 0.05 and 95% confidence intervals (CIs) were employed as the criterion for statistical significance.

###  Machine Learning Algorithm

 The study sample was randomly partitioned, with 70% (excluding those with missing data in the dependent variable) used as the training data to predict MASLD risk. The remaining 30% of the dataset (excluding those with missing data in the dependent variable) was utilized to validate the performance of the proposed algorithms. Each patient was assigned to either the training or validation sets. Four separate ML models were developed by including demographic, clinical, and laboratory information. The models included logistic regression (LR), Naïve Bayes (NB), support vector machine (SVM), and light gradient-boosting machine (LightGBM).^35-38^

 In our study, the transformation of prediction scores into binary outcomes (1 for MASLD and 0 for non-MASLD) was a critical step in evaluating the diagnostic criteria. For LR, the probability output was utilized, which ranges from 0 to 1. A threshold value was selected to convert these probabilities into binary classifications. Initially, a threshold of 0.5 was considered standard practice. This approach was consistently applied to other ML models, such as NB, SVM, and LightGBM. The final binary classifications were validated and evaluated using sensitivity, specificity, positive predictive value (PPV), negative predictive value (NPV), and overall accuracy (ACC), ensuring the reliability and clinical relevance of our models’ predictions.

###  Tune Parameters

 The hyperparameters of each ML model were tuned to improve prediction performance. SVM optimization entailed adjusting the kernel type, regularization parameter, and kernel coefficients. Additionally, the LightGBM was optimized for characteristics including learning rate, tree depth, and minimal data in leaves.^39,40^

###  Performance Evaluation 

 The four models were evaluated using a k-fold cross-validation technique (k = 5) within the validation dataset, followed by an examination of relevant criteria such as the AUC, SE, SP, PPV, NPV, and ACC.^41^ These metrics were formulated as follows:


$$SE=\frac{True\;Positive}{True\;Positive+False\;Negative}$$

$$SP=\frac{True\;Negative}{True\;Negative+False\;Positive}$$

$$PPV=\frac{True\;Positive}{True\;Positive+False\;Positive}$$

$$NPV=\frac{True\;Negative}{True\;Negative+False\;Negative}$$

$$ACC=\frac{True\;Positive+True\;Negative}{Total\;Population}$$

To compare the performance of the four models, the AUC was used as a threshold-independent metric for discrimination. As a result, the best predictive model was established based on the highest AUC value.

###  Variable Importance and Variable Selection 

 Variable identification necessitated a dual strategy that included statistical approaches and clinical factors. The statistical part included the use of mean decrease Gini (MDG) in the RF approach for selecting critical variables. The MDG approach, when integrated into an RF algorithm, enhances the discovery of important variables by aggregating the cumulative decrease in Gini impurity at each tree node split.^42-44^

 In addressing redundant information within our dataset, such as variables presented in both continuous and categorical forms (e.g., continuous systolic blood pressure [SBP] vs. categorized SBP), a systematic approach was employed for variable selection. Initially, both forms were assessed for their relevance and statistical association with MASLD. The univariate analysis was conducted using t-tests and chi-square tests for continuous and categorical variables, respectively, to determine their significance. Redundant variables were then evaluated for correlation, and highly correlated variables were considered for exclusion to minimize multicollinearity. Furthermore, the RF algorithm’s MDG metric was utilized to ascertain the importance of variables in predicting MASLD. The final decision on retaining a variable format was based on a combination of its statistical significance, contribution to the model’s performance, and clinical relevance. Typically, the form that provided more detailed or clinically interpretable information was preferred, ensuring a robust and meaningful predictive model.

## Results

 Participants without ultrasound results were excluded from the study. The excluded sample included 2363 (60.0%) females and 1,575 (40.0%) males, with a mean (SD) age of 48.60 (10.70) and a mean (SD) BMI of 23.31 (4.10). Conversely, data from 6180 subjects with ultrasound results in the baseline phase of the Fasa cohort study underwent analysis. This sample consisted of 3255 (52.7%) females and 2,925 (47.3%) males with a mean (SD) age of 48.42 (9.53), ranging from 35 to 87. Additionally, the mean (SD) BMI of the sample was 24.42 (5.25). There was no significant difference in these variables between the groups with and without ultrasound results. Table 1 provides the descriptive statistics as well as the results of univariate tests for comparing different characteristics of the sample based on the presence of MASLD. Considering the significance level of 10%, all of the variables were statistically related to the presence of MASLD according to these univariate tests.

 After standardizing quantitative variables, the univariate LR model was employed to evaluate the prediction power of each variable solely. Considering the AUC as the most important criterion for assessing the predictive power, it was concluded that waist circumference (AUC = 0.91), BMI (AUC = 0.90), hip circumference (AUC = 0.86), and wrist circumference (AUC = 0.73) had the highest power for predicting MASLD among the variables under study.

**Figure 2:**
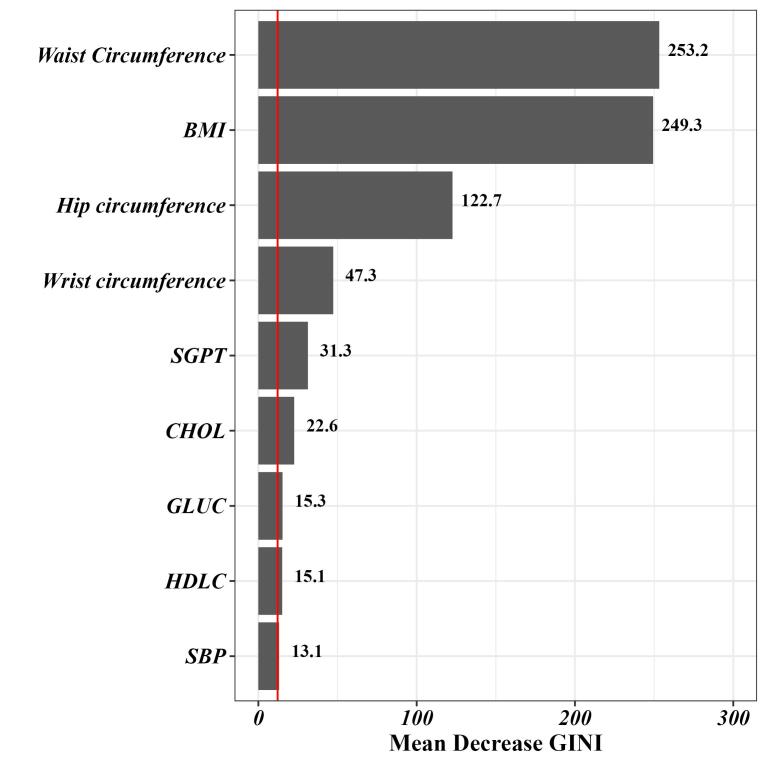
Variable Importance in Predicting MASLD Using the Random Forest Method. *Note*. MASLD: Metabolic dysfunction-associated steatotic liver disease

 Then, an RF algorithm was applied to accurately screen the predictors. In this context, the MDG index was utilized to extract the most influential predictors of MASLD. Figure 2 displays variables with the highest MDG index. In this regard, variables such as waist circumference, BMI, hip circumference, the wrist circumference, serum glutamate pyruvate transaminase (SGPT), cholesterol, glucose (GLUC), high-density lipoprotein cholesterol (HDLC), and SBP had the highest importance in predicting MASLD, respectively.

**Figure 3:**
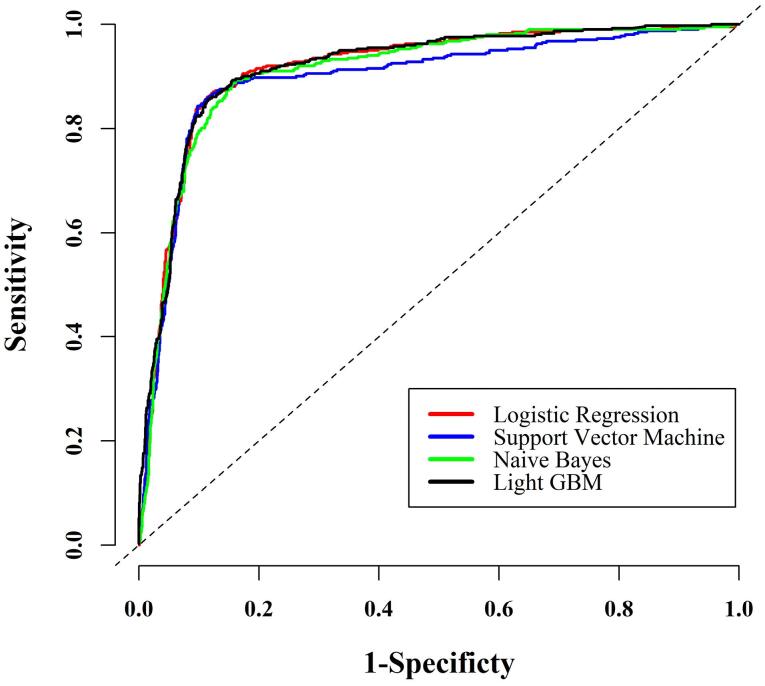
ROC Curves for MASLD Prediction Across All Models. *Note*. ROC: Receiver operating characteristic; MASLD: Metabolic dysfunction-associated steatotic liver disease

 Next, the extracted variables from the previous step (RF method) were used in fitting different ML approaches to find the most powerful model for predicting MASLD in this sample. Table 2 presents the predictive power indices estimated from fitting these models. Regarding all the estimated predictive power indices, the multivariable LR model had the highest power for predicting MASLD among the applied approaches. To graphically compare the predictive power of the ML methods, the estimated AUCs of these techniques are depicted in Figure 3.

**Table 2:** Prediction Performance of Models for MASLD Using Random Forest Feature Selection

**Weighted Models**	**AUC (95% CI)**	**SE (95% CI)**	**SP (95% CI)**	**PPV (95% CI)**	**NPV (95% CI)**	**ACC (95% CI)**
Logistic selected variables (LR)	0.92 (0.90, 0.93)	0.86 (0.82, 0.89)	0.89 (0.87, 0.90)	0.68 (0.64, 0.72)	0.96 (0.95, 0.97)	0.88 (0.86, 0.89)
LightGBM	0.92 (0.90, 0.93)	0.86 (0.82, 0.89)	0.89 (0.87, 0.90)	0.67 (0.63, 0.71)	0.96 (0.94, 0.97)	0.88 (0.86, 0.89)
Naïve Bayes	0.91 (0.89, 0.93)	0.89 (0.86, 0.92)	0.84 (0.82, 0.86)	0.61 (0.57, 0.64)	0.97 (0.95, 0.97)	0.85 (0.83, 0.87)
Support vector machine	0.90 (0.88, 0.92)	0.84 (0.80, 0.88)	0.90 (0.89, 0.92)	0.70 (0.66, 0.74)	0.95 (0.94, 0.96)	0.89 (0.87, 0.90)

*Note*. AUC: Area under the curve; CI: Confidence interval; SE: Sensitivity; SP: Specificity; PPV: Positive predictive value; NPV: Negative predictive value; ACC: Accuracy; LightGBM: Light gradient-boosting machine; MASLD: Metabolic dysfunction-associated steatotic liver disease; LR: Logistic Regression. The variables utilized in all models were selected using the feature selection method of random forest, based on the highest mean decrease in the Gini index as the criterion for selection.

 To present a straightforward interpretation of the predictors in the best model from the previous step, the estimated crude and adjusted odds ratios from fitting univariate and multivariable LR models are reported in Table 3. For instance, a 1 cm increase in waist circumference raises the odds of having MASLD by 17% and 11%, respectively, in the univariate and multivariable LR models.

**Table 3:** Results of Univariate and Multivariable Logistic Regression Analyses to Predict MASLD

**Variables**	**Univariate**		**Multivariable**		
	**OR (95% CI)**	***P*** ** Value**	**Coefficient**	**OR (95% CI)**	***P*** ** Value**
BMI	1.44 (1.42, 1.47)	< 0.001	0.160	1.16 (1.11, 1.21)	< 0.001
CHOL	1.01 (1.01, 1.02)	< 0.001	0.005	1.01 (1.01, 1.01)	< 0.001
GLUC	1.01 (1.01, 1.01)	< 0.001	0.007	1.01 (1, 1.01)	< 0.001
HDLC	0.98 (0.97, 0.98)	< 0.001	-0.005	0.99 (0.98, 0.99)	< 0.001
Hip circumference	1.2 (1.18, 1.21)	< 0.001			
SGPT	1.04 (1.04, 1.05)	< 0.001	0.021	1.02 (1.02, 1.03)	< 0.001
SBP	1.02 (1.02, 1.03)	< 0.001	0.005	1.01 (1, 1.01)	< 0.001
Waist circumference	1.17 (1.16, 1.18)	< 0.001	0.090	1.10 (1.09, 1.12)	< 0.001
Wrist circumference	2.05 (1.95, 2.16)	< 0.001			
Constant	—		-14.610		

*Note*. MASLD: Metabolic dysfunction-associated steatotic liver disease; BMI: Body mass index; CHOL: Cholesterol; GLUC: Glucose; HDLC: High-density lipoprotein cholesterol; SGPT: Serum glutamate pyruvate transaminase; SBP: Systolic blood pressure; CI: Confidence interval; OR: Odds ratio. Multivariable logistic regression was employed, followed by the application of a backward stepwise procedure, to identify the optimal subset of statistically significant variables.

## Discussion

 MASLD is a prevalent liver disease that affects individuals worldwide and is responsible for the majority of cases of liver cirrhosis and cancer. Unfortunately, there is currently no effective medication for MASLD, making early detection and prevention the most powerful strategies for managing the disease.^45^ In this study, as part of a cohort study in the Iranian population, the RF algorithm was employed to identify key variables among 103 body compositions and laboratory indicators for predicting MASLD. In this regard, the top nine variables were selected to fit the ML models. Interestingly, waist circumference and BMI were found to have the highest relative importance among the studied variables. Subsequently, hip circumference, wrist circumference, SGPT, and CHOL were identified as significantly influential in predicting MASLD. Using these selected variables and the data from 6,180 subjects, four ML models were developed for predicting MASLD. Among these models, the LR model stood out with an accuracy of 0.88 and a rather perfect AUC of 0.92.

 A detailed analysis of the available FASC data highlights the importance of considering waist circumference and BMI as crucial factors in evaluating the risk of MASLD. Serving as primary indicators of MASLD, an increase in waist circumference and BMI can remarkably raise the likelihood of developing MASLD. It is notable that based solely on these factors, individuals can be effectively categorized as either having or not having MASLD with an accuracy exceeding 0.80. Furthermore, the AUC for waist circumference and BMI was 0.91 and 0.90, respectively, aligning well with the findings of prior studies in the field. For example, a cross-sectional study conducted by Eslami et al in 2022, involving 71 Iranian women, assessed the predictive value of BMI, waist circumference, and visceral fat for diagnosing MASLD. In their study, BMI was identified as a strong predictor (AUC: 0.78), closely followed by waist circumference (AUC: 0.71).^46^ While their findings demonstrated a notable level of accuracy for BMI and waist circumference, the variation in accuracy values between their study and ours could be linked to the difference in sample sizes, as their study had a smaller sample size compared to ours. In another study performed by Li et al in 2022, involving 8,861 Chinese subjects, the results of a multiple LR model confirmed the significant contribution of BMI and waist circumference to the development of MASLD and revealed that a single unit increase in these variables could substantially raise the risk of MASLD.^47^

 Based on the results of the present research, the third significant factor was hip circumference, which plays a crucial role in evaluating the risk of MASLD. It is worth mentioning that for this particular variable alone, individuals can be categorized as having or not having MASLD with an impressive accuracy rate of 0.81. This discovery is in line with the results of earlier studies conducted in this area. For example, a recent population-based study in Finland conducted by Danielsson et al in 2021 highlighted a strong association between hip circumference and the risk of severe liver disease. Additionally, the findings of the study indicated that combining hip circumference with waist circumference offers a more comprehensive evaluation of an individual’s MASLD risk.^48,49^ Another example pertains to a longitudinal cohort study among adolescents in 2020, where researchers observed that individuals with altered liver elasticity tended to exhibit larger waist and hip circumferences, indicating a heightened level of central adiposity.

 In our study, wrist circumference was the fourth variable highlighted as important in predicting MASLD. To support and validate this finding, it is important to note that our literature review revealed a limited number of studies specifically focusing on assessing body composition factors, particularly wrist circumference, in relation to MASLD.^39,40^ For instance, a study by Razmpour et al on the Iranian population reported that waist circumference, chest circumference, BMI, and trunk fat were significant predictors of MASLD using the RF algorithm, with wrist circumference included in the set of variables.^30^ Their findings also validated the results of our study. It seems that due to the strong predictive power of waist circumference and BMI, wrist circumference tends to have a lower importance, which was not identified as the most influential variable in their study. In the subsequent positions of importance, our findings represented that other factors such as SGPT, CHOL, and GLUC were identified as significant indicators in predicting MASLD. The contribution of these factors has also been supported by several studies.^50-52^

 This study was conducted among rural individuals, and the factors identified were specific to this population. However, it appears that these factors may also influence MASLD in non-rural areas, as indicated by several studies conducted in urban settings reporting these factors as significant. For instance, a study by Amirkalali et al in an urban area of Iran in 2008, involving 5,023 adult individuals, revealed that high waist circumference was a crucial predictor of MASLD.^53^ Therefore, the factors identified in the current study could be considered generally influential factors on MASLD, regardless of whether the area is urban or rural.

 In this study, several advanced ML methods, including LR, SVM, NB, and LGBM, were employed to predict the occurrence of MASLD. These methods have been proven to have a high level of accuracy in predicting MASLD. Notably, LR demonstrated a slight performance advantage compared to the other models. These findings are consistent with those of a study conducted by Yip et al in 2017, which utilized LR, ridge regression, AdaBoost, and decision tree models to predict MASLD in the general population. They also found that both LR and ridge regression displayed the highest accuracy, resulting in an AUC of 0.88.^54^ Similarly, a study by Ma et al examined the use of ML algorithms such as SVM, LR, and RF, using laboratory-based parameters for MASLD diagnosis. In this case, all three methods also demonstrated comparable and high performance in detecting MASLD.^55^ Interestingly, it is noteworthy that the majority of ML studies in this field have mainly focused on laboratory indicators,^27-29^ except for a study conducted by Razmpour et al in 2023. In their research, they utilized ML methods, including a k-nearest neighbor, SVM, radial basis function SVM, Gaussian process GP, RF, neural network, AdaBoost, and NB, for predicting MASLD. Among these methods, RF showed the best performance.^30^ Practically, ML methods have the potential to bring numerous benefits to the classification of medical outcomes and can even be considered alternatives to some medical diagnostic tools such as sonography, ultrasonography, magnetic resonance imaging, and computed tomography scans. While ultrasound is a commonly used method in diagnosing MASLD, it has limitations such as being unable to detect fatty infiltration that is less than 20% steatosis and being operator-dependent.^56-58^ ML methods can help reduce some of these limitations. Utilizing ML techniques on body composition and laboratory factors, which are easier and less time-consuming to obtain, can help physicians make more informed clinical decisions.

 The present study has numerous notable strengths that contribute to its quality. Firstly, our study is one of the first that has used ML methods, based on both body composition and laboratory indicators simultaneously. Additionally, the study benefits from a large sample size, allowing us to identify and employ the most effective prediction model with a high degree of accuracy. Moreover, the participants were carefully selected using the census technique, guaranteeing the inclusion of a substantial portion of eligible individuals within the study. Additionally, unlike most research conducted in this field, the current study provided valuable insights into this particular group by covering a rural population.

 On the other hand, this study had some limitations. Firstly, the results of each patient’s recent sonography played a crucial role in categorizing individuals into two groups (with and without MASLD). It would be more accurate if all individuals underwent sonography examinations conducted by uniformly trained experts at the beginning of the study. The other limitation of this study revolves around the reliance on self-reported data without cross-checking or verification through medical records or other information sources. Furthermore, the age range of the study’s target population, which was limited to individuals between 35 and 70 years old, may restrict the generalizability of the findings to other age groups.

## Conclusion

 This study utilized ML classification models to predict the presence of fatty liver disease based on laboratory and body composition variables, with a focus on rural areas. The results indicated that ML-based decision support systems have the potential to assist physicians in screening, diagnosing, and preventing MASLD. These systems could be especially useful in providing services at a population level and in remote healthcare settings where there is a shortage of trained specialists. Moreover, understanding the level of significance attributed to every predictor, particularly key indicators such as waist circumference and BMI, can greatly assist in the prevention, diagnosis, and treatment of MASLD.

## Supplementary Files

Supplementary file 1 contains Table S1.
