# Supplementary material for: Machine-Learning Application for Predicting Metabolic Dysfunction-Associated Steatotic Liver Disease Using Laboratory and Body Composition Indicators
Source: Arch Iran Med. 2024 Oct 1;27(10):551–62. doi: 10.34172/aim.31269 (PMC11532655; doi:10.34172/aim.31269)
Supplement: Supplementary file 1 — contains Table S1. [file aim-27-551-s001.pdf]

Arch Iran Med. October 2024;27(10):551-S1

10.34172/aim.31269

Supplementary file 1

Table S1. The data source of variables employed in the study.

Variable	Data Source		
	laboratory examinations	Clinical examinations	Questionnaire Surveys
Age (years)			*
Alanine (g/d)			*
ALP (IU/L)	*		*
Alpha Carotene (μ/d)			*
Alpha-Linolenic Acid (ALA) (g/d)			*
Anti_DM_Pill			*
Anti_HTN_Drug			*
Arginine (g/d)			*
Aspartic Acid (g/d)			*
Beta Carotene (mg/d)			*
BMI		*	
BUN (mg/dL)	*		
Caffeine (mg/d)			*
CHOL (mg/dL)	*		
Cholesterol (mg/d)			*
Cryptoxanthin, beta (μ/d)			*
DBP (mmHg)		*	
Docosahexaenoic Acid (DHA) (g/d)			*
Eicosapentaenoic Acid (EPA) (g/d)			*
Fatty Acid (total saturated) (g/d)			*
Fatty Acid (total trans) (g/d)			*
Fatty acids, MUFA (g/d)			*
FH1_Diabetes			*
FH1_Hypertension			*
FH2_Diabetes			*
FH2_Hypertension			*
Fiber (total) (g/d)			*
Fluoride (μ/d)			*
Fructose (g/d)			*
Gender			*
GGT	*		
GLUC (mg/dL)	*		
Glucose (dextrose) (g/d)			*
Glutamic Acid (g/d)			*
Glycine (g/d)			*
GR (%)	*		
Has Diabetes			*
Has Gallstone			*
Has Learning Disability			*
Has Osteoporosis			*
Has Rheumatic Disease			*
Has Shortness of Breath			*
Has Sternum Irritation			*
Has Swelling			*
Has Thyroid Disease			*

Has Weight Loss			*
HCT (Gr/dl)	*		
HDLC (mg/dL)	*		
HGB (Gr/dl)	*		
Hip Circumference (cm)		*	
Histidine (g/d)			*
Isoleucine (g/d)			*
LDL (mg/dL)	*		
Leucine (g/d)			*
Lutein + zeaxanthin (μ/d)			*
LY (%)	*		
Lycopene (μ/d)			*
Lysine (g/d)			*
Magnesium (mg/d)			*
Maltose			*
MCH (Pg)	*		
MCHC (Gr/dl)	*		
MCV (FL)	*		
MET (h/d)			*
Methionine (g/d)			*
MO (%)	*		
n-3 (total) (g/d)			*
n-6 (total) (g/d)			*
Naps (Hour)			*
Night Sleep (Hour)			*
Pantothenic Acid (mg/d)			*
Phenylalanine (g/d)			*
PLT (Cumm)	*		
Potassium (mg/d)			*
Proline (g/d)			*
RBC (Cumm)	*		
Retinol (μ/d)			*
SBP (mmHg)		*	
Selenium (μ/d)			*
Serine (g/d)			*
SGOT (IU/L)	*		
SGPT(IU/L)	*		
Smoking			*
Sugar (g/d)			*
TG	*		
TG_Lowering_Drug			*
Threonine(g/d)			*
Total lipid (g/d)			*
Tryptophan (g/d)			*
Tyrosine (g/d)			*
Use Non Cig Tobacco			*
Using Alcohol			*
Using Insulin			*
Using Statin			*

Valine (g/d)			*
Vitamin A (µ/d)			*
Vitamin B6 (mg/d)			*
Vitamin C (mg/d)			*
Vitamin E (mg/d)			*
Vitamin K (µ/d)			*
Waist circumference		*	
WBC (Cumm)	*		
Wrist Circumference (cm)		*	
WSI_total			*

In the present study, data were collected using three methods: laboratory examinations, clinical examinations, and questionnaire surveys. Table S1 details how each type of data was obtained. In the current study, among many questionnaires completed for participants to obtain baseline information on lifestyle, environmental, and social exposures, a semi-quantitative food frequency questionnaire (FFQ) was developed for use in the PERSIAN Cohort Study, to assess diet's role in non-communicable diseases. Our 125 item FFQ was designed as a semi-quantitative, interviewer-administered questionnaire, enquiring about individuals' usual intake of each food item over the year prior to the interview date. Frequency data obtained for each food item on the FFQs were converted to daily intake, then multiplied by the weight (in grams) of the portion size consumed each time to obtain the grams consumed from each food item per day (grams/day). The USDA Food Composition Tables (USDA-FCT) were used to obtain daily energy and other nutrients intake of food items.

It should be noted that the methodology for the questionnaire used was outlined in the paper titled "Validity and reproducibility of a food frequency questionnaire assessing food group intake in the PERSIAN Cohort Study," published in the Frontiers journal (Frontiers in Nutrition).

Reference

Validity and reproducibility of a food frequency questionnaire assessing food group intake in the PERSIAN Cohort Study - PMC (nih.gov)
